# Treatment with head-lift exercise in head and neck cancer patients with dysphagia: results from a randomized, controlled trial with flexible endoscopic evaluation of swallowing (FEES)

**DOI:** 10.1007/s00520-022-07462-z

**Published:** 2022-12-17

**Authors:** Hans Dotevall, Lisa Tuomi, Kerstin Petersson, Helena Löfhede, Henrik Bergquist, Caterina Finizia

**Affiliations:** 1 Department of Otorhinolaryngology, Head and Neck Surgery, Institute of Clinical Sciences, Sahlgrenska Academy, University of Gothenburg, Gothenburg, Sweden; 2 Department of Otorhinolaryngology, Head and Neck Surgery, Region Västra Götaland, Sahlgrenska University Hospital, Gothenburg, Sweden; 3 Institute of Neuroscience and Physiology, Speech and Language Pathology Unit, Sahlgrenska Academy, University of Gothenburg, Gothenburg, Sweden

**Keywords:** Head and neck neoplasms, Deglutition disorders, Intervention study, Radiotherapy, Randomized

## Abstract

**Background:**

This randomized study aimed to evaluate the effects of the Shaker head-lift exercise (HLE) to improve dysphagia following oncologic treatment for head and neck cancer (HNC).

**Methods:**

Patients with dysphagia following oncologic treatment for HNC were randomly assigned to intervention (*n* = 23) or control (standard dysphagia management, *n* = 24) groups. Swallowing was evaluated at baseline and at 8-week follow-up using flexible endoscopic evaluation of swallowing (FEES) and self-perceived swallowing with the Eating Assessment Tool (EAT-10). Analysis was performed regarding secretion, initiation of swallow, residue after swallowing, and penetration/aspiration.

**Results:**

Few statistically significant differences were found in the FEES analysis. Some improvement of self-perceived swallowing function was found in both groups. Adherence to training was high.

**Conclusions:**

This randomized study regarding the effect of the HLE demonstrated that swallowing outcome measures used in assessment of FEES did not improve in patients treated with radiotherapy for patients with dysphagia following HNC.

## Introduction

Oropharyngeal dysphagia is a common side effect after treatment for head and neck cancer (HNC). Unidentified dysphagia causes significant morbidity, increased mortality, malnutrition, and decreased quality of life [1–5]. Dysphagia may develop or progress years after radiation-based treatment due to fibrosis, muscular atrophy, or cranial neuropathy [6].

Chronic dysphagia has been reported to occur in up to 69% of patients 6–12 months after oncologic treatment for HNC [7, 8]. Impairment of swallowing function may lead to aspiration of liquid or food to the airways [3, 4, 9, 10]. Studies have reported an incidence of aspiration between 16 and 84% in HNC patients [3, 6, 8–10]. Silent aspiration, i.e., passage of liquid or food below the glottis without external signs such as coughing or choking, is prevalent after radiotherapy or chemoradiotherapy for HNC in up to 35% of patients [11]. Aspiration pneumonia may occur as a consequence of chronic aspiration [6, 12].

Previous studies have demonstrated potential positive effects on swallowing function following behavioral treatment in patients with HNC and dysphagia [13, 14]. Still, due to heterogeneity in published studies regarding, for example, types of interventions, timing of treatment, study groups, and outcome measures, there is a need for further investigations of dysphagia treatment in patients with HNC [13–15]. Furthermore, most studies employ several different types of treatment techniques [13, 14], making it difficult to determine the effect of singular treatment methods.

The Shaker head-lift exercise (HLE) has been utilized for dysphagia treatment in patients with HNC in several studies, either as a part of a more extensive treatment program [16–19] or as a specific treatment modality [20, 21]. The effect of HLE on swallowing function has also been studied in stroke patients [22–24] and healthy adults [25, 26].

The Shaker HLE was originally introduced primarily for treatment of impaired upper esophageal sphincter (UES) opening during swallowing [24, 26]. The exercise includes both isometric and isokinetic muscular training aiming at strengthening the suprahyoidal, thyrohyoid, and pharyngeal muscles in order to improve hyoid and laryngeal elevation during deglutition [27]. The rationale for this exercise is thus to increase swallowing efficiency and improve the bolus transit from the pharynx to the esophagus [24, 26, 28].

Previous studies indicate that the Shaker HLE may have a beneficial effect on oropharyngeal swallowing function in HNC patients [20, 21, 27]. In particular, reduction of post-swallow aspiration, better maintained movement of the hyoid bone, thyrohyoid shortening, UES opening, less aspiration during swallowing, and strengthening the suprahyoidal muscles were observed after HLE treatment [20, 21, 27]. The results from a pilot study preceding this randomized study indicated that health-related quality of life and self-reported swallowing function improved after 8 weeks of HLE intervention in patients following stroke or treatment for HNC [29]. The Shaker HLE is feasible and possible to perform for most patients [29]. However, previous analysis of data from the present randomized study of videofluoroscopic examination of swallowing (VFSS) only demonstrated minor changes pertaining penetration and/or aspiration events or structural movement variables during swallowing after HLE treatment in patients with HNC [30]. Further analysis of the effect of the HLE regarding other aspects of swallowing function in HNC patients is needed.

The objective of this randomized study was to investigate the effect of the Shaker HLE on swallowing function using flexible endoscopic evaluation of swallowing (FEES) in patients with HNC treated with radiotherapy with or without concomitant chemotherapy.

## Materials and methods

### Subjects

Adult patients with tumors of the tonsil, base of tongue, hypopharynx or larynx treated with external beam radiation therapy (EBRT) ± brachytherapy or chemotherapy, at Sahlgrenska University Hospital, Sweden, between at least 6 months up to 36 months prior to recruitment were assessed for eligibility in the study. Further inclusion criteria were swallowing difficulties resulting in Penetration Aspiration Scale (PAS) score [31] of ≥ 2 (i.e,. material enters the airway, remains above the vocal folds, and is ejected from the airway) on more than one swallow on the initial VFSS at the time of inclusion and no dysphagia previous to cancer treatment. Exclusion criteria were previous surgery for HNC (except tonsillectomy or diagnostic sample excision), previous RT or other treatment for HNC, tracheostomy, neurological or neuromuscular disease with possible impact on swallowing function, and/or inability to perform the HLE. Patients who were unable to swallow any bolus at all at baseline were also excluded, since no measurement could be made.

The patients were randomized to either active treatment with HLE in combination with standard dysphagia management (intervention group) or standard dysphagia management only (control group). Standard dysphagia management was provided by a speech language pathologist (SLP) according to the clinical routines at the time of the study and included advice about food, drinking, head position, or swallowing maneuvers, such as the supraglottic swallow, effortful swallow, and the Mendelsohn maneuver during meals. The randomization was balanced according to tumor type, T stage, age, gender, comorbidity measured with Adult Comorbidity Evaluation-27 (ACE-27) [32, 33], and the PAS score [31] on VFSS at the time of inclusion.

An 80% power calculation was performed (Mann–Whitney *U* test, alpha = 0.05) prior to study start where a sample size of 25 participants in each group was determined assuming a clinically relevant difference of one point on the PAS score between the study groups, with a standard deviation of 1.2. In order to compensate for possible dropouts, the recruitment aimed to include 30 participants in each group.

The cancer treatment was given according to the regional cancer treatment program. EBRT was delivered as intensity modulated/volumetric modulated radiation therapy (IMRT/VMAT) with specified dose constraints to the parotid gland. The radiotherapy was either conventional (once daily, *n* = 41) or accelerated (twice daily, *n* = 6). The given dose was typically a total of 68 Gy with 2.0 Gy/fractions, once daily, 5 days a week. Chemotherapy was given either as induction (*n* = 29) or concomitant (*n* = 8) therapy. Induction chemotherapy was given to patients with more advanced disease (*n* = 29) and generally consisted of two cycles of cisplatin 100 mg/m^2^ day 1 and 5-fluorouracil 1000 mg/m^2^ day 1 through 5. The cycle interval was 22 days. Concomitant chemotherapy (*n* = 8) generally consisted of six cycles of cisplatin 40 mg/m^2^ once a week. Ten participants received no chemotherapy.

### Intervention

The HLE consists of isometric and isokinetic head lifts in supine position [24, 26]. The exercise included sustained/static head lifts for 60 s three times with 1-min rest between the lifts (isometric training). This was followed by 30 consecutive repetitions of head lifts (isokinetic training). The exercise was performed three times daily during a period of 8 weeks, according to the treatment scheme. Subjects were instructed individually on how to perform the HLE by an SLP. The subjects also received written and video instructions. During the first 2 weeks, the subjects were assisted by an SLP during three exercise sessions. From the third week, the SLP assisted at one exercise session every 2 weeks and performed follow-ups by telephone in between. The participants in the control group did not receive any SLP contact during the trial period. All study SLPs were instructed by written, video, and oral instruction of the HLE, to ensure consistency to the delivery of the intervention.

The subjects in the intervention group documented the amount of training and, where necessary, stated reasons for not completing the exercise in an exercise diary.

### Assessment

#### Eligibility for inclusion using videofluoroscopic examination of swallowing

A VFSS assessing the eligibility for inclusion in the study was performed. Patients were presented with different amounts and consistencies of barium contrast, similar to the protocol described below. Details of the VFSS have been described elsewhere [30]. A gastrointestinal radiologist and an SLP scored the video recordings of the VFSS according to PAS [31] prior to inclusion to assess which patients were eligible for inclusion in the study.

#### Flexible endoscopic evaluation of swallowing

The FEES was performed by a study SLP at baseline and after the 8 weeks of intervention (before and after intervention) based on the procedure described by Langmore et al. [34]. A flexible endoscope was passed through the nose to obtain an overview over the pharynx and larynx. Different equipment was used throughout the study; Olympus ENF-P4 flexible endoscope (Olympus Inc., Japan) attached to a Wolf-Type 5052 light source (Richard Wolf GmbH, Germany), Olympus ENF-VH video fiber endoscope attached to an Olympus Elite II OTV-S200 light source (Olympus Inc.), or Xion EV-NC videofiberendoscope attached to a Xion Endoportable CFT-003 dock (Xion GmbH, Germany). Digital video recordings were made using a video database system (IMIS, Atea AB, Sweden) or, in a few cases, a Xion Endoportable CFT-003 or by digitization of analogue VHS recordings. Prior to endoscopy, the nasal mucosa on the most patent side was decongested and anesthetized locally with lidocaine 3.4%/naphazoline 0.02% solution using cotton attached to a thin feeding catheter (Unomedical Purifeed, CH 06, Denmark) in all subjects, in order to reduce the discomfort. Care was taken not to anesthetize the pharyngeal mucosa.

Boluses with different consistencies and volumes colored with green caramel color were presented according to the following protocol: 5 ml mildly thick liquid (corresponding to International Dysphagia Diet Standardisation Initiative (IDDSI), level 2 [35]); 3, 10, and 20 ml of thin liquid (corresponding to IDDSI level 0); and one-fourth soft biscuit (corresponding to IDDSI level 7). All boluses were swallowed on command, except for the 20 ml thin liquid bolus which the subjects were allowed to drink at a self-determined pace, with one trial per bolus. The mildly thick liquids and the 3 ml thin liquid were administered with a spoon. Ten- and 20-ml thin liquid were given in a cup. In order to ensure that the colored boluses were the same consistencies throughout the study, all colored consistencies were according to the IDDSI protocol [36]. The patients did not use any of the recommended maneuvers or advice during the FEES examination used in the analysis. The investigators were allowed to exclude boluses during the FEES examination if they considered that there was a risk of harmful aspiration; for example, if the participant aspirated without being able to clear the airway, the larger amount of the same consistency was not tested, or if the patient had difficulties chewing, the biscuit was not given. If a bolus was excluded at the first examination, it could be tested again at follow-up, if the investigators deemed it safe.

### Analysis of flexible endoscopic evaluation of swallowing

Two SLPs with more than 5-year experience of dysphagia diagnosis and treatment performed the blinded analysis of the FEES examinations individually; the judges were not otherwise involved in the study. The video recordings were edited (i.e., personal data were removed in order to de-identify the recording) and presented in a randomized order on an iPad (Apple Inc., USA) without information about the patients or timepoints in the study. Both judges evaluated all recordings. Twenty percent (19 of 94) of the videos were duplicated by randomization for analysis of intra-rater reliability. Thus, a total of 113 videos were evaluated. A 2-day training session was undertaken before the evaluation in order to improve evaluation consistency between the judges.

To enable an overall evaluation of swallowing function using FEES in this cohort, the selection of variables in the FEES assessment was based on relevant physiological features and choice of assessment scales with sufficient validity as described in published literature. The following FEES variables were evaluated:

1. Secretions in the pharynx and larynx before the first bolus using the Murray secretion scale, a four-grade scale (1 = no visible or some transient bubbles of secretion in the vallecula and hypopharynx, 2 = deeply pooled secretion in the vallecula and sinus pyriformis, 3 = any secretion that changed from a “2” to a “4” rating during the observation, and 4 = secretion in the laryngeal vestibule) [37, 38].
2. Initiation of the pharyngeal swallow for all boluses. The position of the bolus head at the initiation of the swallow was assessed according to a four-grade scale (1 = initiation at the upper epiglottis, 2 = exceeds epiglottis or immediate initiation at pyriform sinus, 3 = initiation at pyriform recess, and 4 = no initiation of swallowing [39].
3. PAS applied for FEES for all boluses [31, 40, 41]. The PAS includes eight scale steps where “1” denotes no material entering the airway and “8” material enters the airway, passes below the vocal fold, and no effort is made to eject. The PAS was assessed as illustrated in the previously published report on VFSS data [30].
4. Residue in the vallecula and pyriform sinuses, respectively, after each bolus according to the Yale Pharyngeal Residue Severity Scale [42, 43]. This is a five-grade scale where 1 = no residue, 2 = trace coating of residue, 3 = epiglottic ligament visible or quarter full pyriform sinuses (mild), 4 = epiglottic ligament covered or half full pyriform sinuses (moderate), and 5 = filled to epiglottic rim or up to aryepiglottic folds (severe).
5. Swallowing Performance Scale (SPS) [44]. This is a seven-grade global assessment scale of swallowing performance where 1 indicates normal swallowing and 7 indicates severe impairment. The evaluation of the SPS was made at the end of each FEES examination, using the information available from the FEES.

### Patient-reported outcome

For subjective self-evaluation of swallowing function, all subjects answered a Swedish version of the questionnaire Eating Assessment Tool (EAT-10) before and at the 8-week follow-up. EAT-10 is a ten-item instrument reflecting different aspects of swallowing dysfunction. Each item is estimated on a five-point scale (0 = no problem, 4 = severe problem). The maximum score is thus 40 points. Normative data indicate that a score of ≥ 3 can be regarded as abnormal [45]. The EAT-10 has been used in HNC, and results have been shown to correspond well to functional eating during and after chemoradiotherapy [46].

### Statistical analysis

All statistical analyses were performed using SAS version 9.4. All tests performed were two-tailed non-parametric tests, with the significance level set to *p* < 0.05. For continual variables, the mean, standard deviation, median, and range are presented for descriptive purposes. Number and percentages are presented for categorical variables.

Comparisons between groups were performed using Fisher’s exact test for dichotomous variables (sex, feeding tube at baseline), the Mantel–Haenszel chi-squared test for ordered categorical variables (tumor stage, comorbidity), chi-squared test for non-ordered categorical variables (smoking, tumor localization, xerostomia, standard dysphagia management), and the Mann–Whitney *U* test for continuous variables.

Comparison of the outcome variables between groups before and after treatment was analyzed using the Mann–Whitney *U* test. For comparison of changes in scores within groups before versus after intervention, the Wilcoxon signed-rank test was used. For the differences of changes between the groups, a 95% confidence interval of the Fisher non-parametric permutation test was calculated. Effect sizes were calculated to further determine the magnitude of group differences. Effect size was calculated at the absolute difference in mean divided by the pooled standard deviation (SD), and interpreted using Cohen’s standard criteria: trivial (0 to < 0.2), small (0.2 to < 0.5), moderate (0.5 to < 0.8), and large (≥ 0.8) [47].

As for the results of the FEES evaluations, the two raters rated all samples independently. When the sample was rated identically of both raters, that number was used; however, if their rating differed, the mean values of the two judges for each rating were used. The reliability within (intra-rater) and between (inter-rater) the judges was calculated using exact agreement in percent, agreement within one scale step in percent, and weighted kappa statistics [48]. Landis and Koch characterized kappa values < 0 as indicating no agreement and 0–0.20 as slight, 0.21–0.40 as fair, 0.41–0.60 as moderate, 0.61–0.80 as substantial, and 0.81–1 as almost perfect agreement [49].

### Ethical considerations

The study was approved by the Regional Ethical Review Board in Gothenburg, Sweden and was conducted according to the Declaration of Helsinki. Before inclusion in the study, all participants gave their written informed consent. The study population has been described in part, in previous work [30].

## Results

**Fig. 1:**
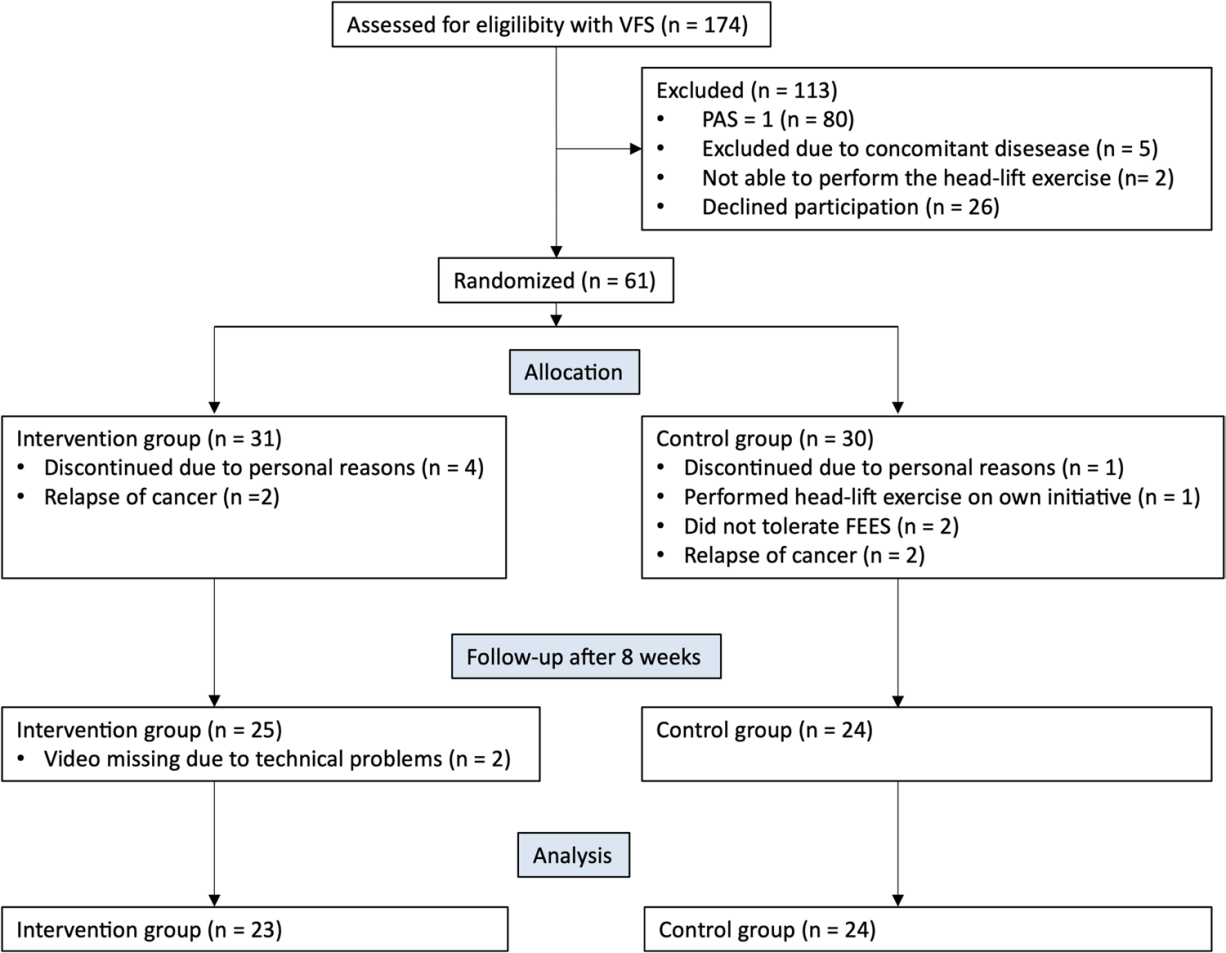
Trial overview. PAS denotes the Penetration Aspiration Score on the initial examination with videofluoroscopic examination of swallowing (VFSS). FEES denotes flexible endoscopic examination of swallowing

An overview of the trial is given in Fig. 1. One hundred and seventy-four individuals were assessed for eligibility, of which 61 were included in the study. Forty-seven were eligible for analysis, 23 in the study group and 24 in the control group (Fig. 1). Fourteen patients were not included in the analysis, eight in the study group and six in the control group, and were therefore considered as dropouts, reasons listed in Fig. 1. Dropout analysis revealed no significant differences between the subjects who completed the study and the dropouts. However, there was a tendency towards a higher proportion of smokers (*p* = 0.058) and subjects with a higher comorbidity score (*p* = 0.077) among the dropouts.

Sociodemographic and clinical data of the study participants are presented in Tables 1 and 2. There were no statistically significant differences regarding age, gender, body mass index (BMI), tumor localization, cancer treatment, time since end of radiotherapy, other types of intervention for dysphagia, comorbidity, trismus, salivary flow and xerostomia, or smoking habits between the groups. Feeding tube use revealed no statistically significant difference between the groups (*n* = 1 in the intervention group, *n* = 3 in the control group), and no changes regarding feeding tube use occurred during the follow-up period. Furthermore, there were no significant differences between the two study groups regarding chemotherapy or radiation dose.

**Table 1:** Demographics of the subjects included in the analysis

Variable	Intervention group (no. of patients = 23)	Control group (no. of patients = 24)	*p* value
	Mean (SD)	Mean (SD)	
Age	63.0 (8.2)	62.7 (6.4)	0.67
BMI (kg/m^2^)	21.3 (7.1)	22.2 (7.3)	0.50
Time since end of radiotherapy (months)	11.4 (6.1)	13.0 (8.2)	0.81

BMI = body mass index

**Table 2:** Patient demographic continued

Variable	Intervention group (no. of patients = 23)	Control group (no. of patients = 24)	*p* value
	No. of patients (%)^†^	No. of patients (%)^†^	
Sex			
Male	16 (70)	19 (79)	0.67
Female	7 (30)	5 (21)	
Smoking			
Never smoked	7 (30)	6 (25)	0.73
Quit smoking ≥ 12 months ago	10 (44)	14 (58)	
Quit smoking < 12 months ago	4 (17)	3 (13)	
Smoker	2 (9)	1 (4)	
Tumor localization			
Tonsil	10 (43)	10 (42)	0.72
Tongue base	9 (39)	9 (38)	
Larynx	3 (13)	3 (13)	
Hypopharynx	1 (4)	2 (8)	
Tumor stage			
I	1 (4)	2 (8)	0.94
II	4 (17)	2 (8)	
III	0 (0)	2 (17)	
IV	18 (78)	18 (67)	
Comorbidity (ACE 27)			
None	12 (52)	9 (38)	0.17
Mild	10 (43)	11 (46)	
Moderate	1 (4)	4 (17)	
Feeding tube at baseline	1 (4)	3 (13)	0.64
Xerostomia			
None	3 (13)	0 (0)	0.44
Mild	1 (4)	3 (12)	
Moderate	9 (39)	11 (44)	
Severe	10 (44)	10 (42)	
Standard dysphagia management			
Advice about food	15 (65)	18 (75)	0.51
Advice about drinking	9 (39)	13 (54)	0.38
Head position	4 (17)	4 (17)	1.00
Swallowing maneuver	2 (9)	2 (8)	1.00
Other swallowing advice^‡^	6 (26)	9 (38)	0.53

*ACE-27* Adult Comorbidity Evaluation-27, *PEG* percutaneous endoscopic gastrostomy

^†^Percentages rounded, therefore does not always sum to 100

^‡^Other advice included drinking/eating in small sips/bites, eating/drinking slowly, swallowing repeatedly, and taking small sips between bites

All subjects in both groups were offered dysphagia care by SLPs according to the standard routines at the time of the study. The type of standard treatment given is described in Table 2. There was no significant difference between the groups regarding concomitant dysphagia treatment.

According to the training diaries, the subjects in the study group performed in mean between 80–92% of the isokinetic and isometric training per week during the period of 8 weeks (Table 3). Reasons for not being able to perform or complete a whole exercise session were listed in the diary. Some participants reported muscle soreness or pain after the exercise, but no serious side effects were reported.

**Table 3:** Adherence to treatment over the 8 intervention weeks according to exercise diaries in the intervention group, reported as % of recommended exercise and mean number of exercises per day

	Adherence to training per week							
	1	2	3	4	5	6	7	8
Percent of isometric training	79.1	85.2	85.9	90.7	93.1	92.5	90.5	80.4
Percent of isokinetic training	81.5	88.4	85.7	88.6	90.8	90.7	88.5	78.1
Mean number of training sessions per day	2.6	2.7	2.7	2.8	2.9	2.8	2.8	2.5

### Penetration aspiration scores

At baseline, the mean values of worst PAS scores were 3.9 and 4.5 in the intervention and control group, respectively. A total of 12 participants (26%) demonstrated a worst PAS of ≥ 6 (i.e., aspiration) at baseline (*n* = 5 in the intervention group, *n* = 7 in the control group). At follow-up, the corresponding number was 7 (15%), 2 were in the intervention group and 5 in the control group. No statistically significant differences in PAS were observed between the intervention and the control group at baseline or follow-up (Table 4). Two statistically significant within-group changes were found: improvement of PAS regarding 5 ml thick liquid in the intervention group and 3 ml thin liquid in the control group. The differences of changes between the groups revealed no statistically significant differences, and only trivial to small effect sizes.

**Table 4:** Comparison of the worst penetration aspiration scores (PAS) and PAS for different consistencies and volumes between the intervention and control groups using results from the flexible endoscopic evaluation of swallowing at baseline and follow-up

	Intervention group (*n* = 23)				Control group (*n* = 24)				Differences between groups		Differences of changes between groups	Difference between groups	Effectsize^†^
	Baseline	Follow-up	Difference	Within-group difference	Baseline	Follow-up	Difference	Within-group difference	Baseline	Follow-up			
	Mean (SD) Median (min; max)	Mean (SD) Median (min; max)	Mean (95% CI) Median (min; max)	*p* value	Mean (SD) Median (min; max)	Mean (SD) Median (min; max)	Mean (95% CI) Median (min; max)	*p* value	*p* value	*p* value	*p* value	Mean (95% CI)	
Worst PAS^‡^ overall	3.9 (2.1) 4 (1; 7.5)	3.8 (2.0) 4 (1; 8)	0.0 (− 1.1; 1.0) 0 (− 6; 4)	1.00	4.6 (1.9) 5 (1; 8)	4.2 (2.1) 4 (1; 8)	− 0.3 (− 1.3; 0.7) 0 (− 4.5; 5)	0.52	0.24	0.62	0.68	− 0.3 (− 1.7; 1.0)	0.13
Thick 5 ml	1.9 (1.3) 1 (1; 5)	1.7 (1.1) 1 (1; 5)	− 0.1 (− 0.5; 0.2) 0 (− 2; 2)	0.53	2.2 (1.2) 2 (1; 5)	1.6 (0.9) 1 (1; 3)	− 0.5 (− 0.8; − 0.1) 0 (− 2; 0.5)	0.017	0.29	0.63	0.21	− 0.3 (− 0.8; 0.2)	0.41
Thin 3 ml	2.6 (2.2) 1 (1; 7.5)	2.0 (1.4) 1 (1; 5)	− 0.7 (− 1.3; − 0.1) 0 (− 4; 1)	0.033	2.4 (1.8) 2 (1; 8)	1.9 (1.0) 2 (1; 4.5)	− 0.6 (− 1.5; 0.4) 0 (− 7; 3.5)	0.25	0.90	0.91	0.90	0.1 (− 1.0; 1.2)	0.05
Thin 10 ml	2.9 (1.9) 2.5 (1; 7)	2.7 (1.8) 2 (1; 6)	− 0.2 (− 1.1; 0.7) 0 (− 6; 4)	0.69	3.2 (1.7) 3 (1; 7)	3.0 (1.6) 3 (1; 6)	− 0.3 (− 1.4; 0.8) 0 (− 4; 4)	0.60	0.48	0.58	0.93	− 0.1 (− 1.4; 1.3)	0.04
Thin 20 ml	3.5 (1.8) 3.5 (1; 7)	3.4 (1.9) 3 (1; 7)	− 0.1 (− 1.1; 1.0) 0 (− 4; 4)	0.96	4.1 (2.1) 4 (1; 7.5)	3.9 (2.3) 4 (1; 8)	− 0.1 (− 1.4; 1.3) − 0.5 (− 4.5; 5.5)	0.88	0.34	0.61	0.96	− 0.1 (− 1.7; 1.5)	0.03
Biscuit	1.8 (1.1) 1 (1; 4.5)	2.3 (2.1) 1 (1; 8)	0.6 (− 0.1; 1.2) 0 (− 1; 5)	0.13	2.0 (1.2) 2 (1; 5)	2.0 (1.0) 2 (1; 5)	− 0.1 (− 0.6; 0.5) 0 (− 2; 4)	0.89	0.67	0.52	0.18	− 0.6 (− 1.5; 0.3)	0.44

*CI* confidence interval, *PAS* Penetration Aspiration Scale, *SD* standard deviation

^†^Effect size criteria: trivial (0 to < 0.2), small (0.2 to < 0.5), moderate (0.5 to < 0.8)**,** and large (≥ 0.8)

^‡^PAS (1**–**8): 1 indicates swallowing without penetration/aspiration. A higher score indicates worse swallowing function. PAS is reported from two raters; if there was a discrepancy between the raters, the mean of the ratings is reported**;** therefore**,** the PAS can assume values between whole scale steps

### Analysis of swallowing function

No differences were found between the groups at baseline or follow-up regarding secretions before the first bolus, initiation of the swallow, post-swallow residue, and SPS (Table 5). At the 8-week follow-up, there was a statistically significant difference regarding the initiation for swallowing of biscuit, where the intervention group demonstrated better initiation (mean value 1.0 vs. 1.1, *p* = 0.041). However, the values did not change significantly before vs. after treatment in any of the groups, and the only statistically significant differences regarding the changes was found when comparing vallecular residue for 3 ml thin liquid, where the intervention improved slightly, while the control group deteriorated (mean change − 0.2 and 0.2, respectively, *p* = 0.049). Effect sizes were mainly trivial to small, but with a moderate effect size when comparing the differences of the intervention and control group regarding vallecular residue for 3 ml thin liquid.

**Table 5:** Results of the analysis of the flexible endoscopic evaluation of swallowing at baseline reporting secretion before swallow, initiation of swallow, residue, Swallowing Performance Scale, and EAT-10 scores in intervention and control groups at baseline and follow-up

	Intervention group (*n* = 23)				Control group (*n* = 24)				Differences between groups				
	Baseline	Follow-up	Difference	Within-group difference	Baseline	Follow-up	Difference	Within-group difference	Baseline	Follow-up	Change baseline to follow-up		
	Mean (SD) Median (min; max)	Mean (SD) Median (min; max)	Mean (95% CI*) Median (min; max)	*p* value	Mean (SD) Median (min; max)	Mean (SD) Median (min; max)	Mean (95% CI*) Median (min; max)	*p* value	*p* value	*p* value	*p* value	Mean (95% CI*)	Effect size†
Secretion before swallow^‡§^	2.0 (1.2) 1.5 (1; 4)	1.8 (1.0) 1.5 (1; 4)	− 0.3 (− 0.9; 0.3) 0 (− 3; 2.5)	0.35	1.9 (1.1) 1.5 (1; 4)	2.1 (1.2) 1.5 (1; 4)	0.2 (− 0.4; 0.8) 0 (− 2.5; 3)	0.55	0.73	0.41	0.26	0.5 (− 0.3; 1.3)	0.345
Initiation of swallow^‡§^													
Thick 5 ml	1.0 (0.1) 1 (1; 1.5)	1.1 (0.3) 1 (1; 2)	0.1 (0.0; 0.2) 0 (0; 1)	0.13	1.3 (0.5) 1 (1; 3)	1.2 (0.4) 1 (1; 2)	0.1 (− 0.1; 0.2) 0 (− 0.5; 1)	0.56	0.31	0.86	0.81	0.0 (− 0.2; 0.1)	0.142
Thin 3 ml	1.3 (0.6) 1 (1; 3)	1.35 (0.63) 1 (1; 3)	0.1 (− 0.3; 0.4) 0 (− 2; 2)	0.81	1.4 (0.7) 1 (1; 3)	1.4 (0.7) 1 (1; 3)	0.1 (− 0.1; 0.3) 0 (− 0.5; 1.5)	0.38	0.81	0.77	0.93	0.0 (− 0.4; 0.5)	0.061
Thin 10 ml	1.4 (0.7) 1 (1; 3)	1.4 (0.7) 1 (1; 3)	0.0 (− 0.3; 0.2) 0 (− 2; 1)	0.99	1.3 (0.6) 1 (1; 3)	1.4 (0.5) 1.3 (1; 3)	0.1 (− 0.1; 0.3) 0 (− 1; 1)	0.47	0.44	0.44	0.58	0.1 (− 0.2; 0.5)	0.222
Thin 20 ml	1.4 (0.6) 1 (1; 3)	1.6 (0.6) 1.5 (1; 3)	0.2 (− 0.3; 0.6) 0.5 (− 2; 1.5)	0.45	1.3 (0.6) 1 (1; 3)	1.4 (0.5) 1 (1; 2)	0.1 (− 0.2; 0.3) 0 (− 1; 1)	0.72	0.83	0.48	0.70	− 0.1 (− 0.6; 0.4)	0.151
Biscuit	1.1 (0.2) 1 (1; 2)	1.0 (0.0) 1 (1; 1)	0.0 (− 0.1; − 0.0) 0 (− 1; 0)	1.00	1.1 (0.3) 1 (1; 2)	1.1 (0.4) 1 (1; 2.5)	0.0 (− 0.2; 0.2) 0 (− 1; 1.5)	1.00	0.18	0.041	0.84	0.0 (− 0.2; 0.3)	0.129
Residue in vallecula^‡§^													
Thick 5 ml	2.7 (0.6) 2.5 (1.5; 4)	2.8 (0.7) 3 (1.5; 4)	0.1 (− 0.2; 0.3) 0 (− 0.5; 1)	0.58	2.9 (0.8) 2.5 (2; 4.5)	2.8 (0.8) 3 (1.5; 4.5)	− 0.1 (− 0.4; 0.2) 0 (− 1; 1)	0.49	0.90	0.87	0.31	− 0.2 (− 0.5; 0.1)	0.343
Thin 3 ml	2.4 (0.4) 2.5 (1.5; 3.5)	2.2 (0.5) 2 (1; 3)	− 0.2 (− 0.4; 0.1) 0 (− 1.5; 1)	0.20	2.3 (0.5) 2.25 (1.5; 3)	2.4 (0.5) 2.5 (2; 4)	0.2 (− 0.1; 0.4) 0 (− 0.5; 1.5)	0.20	0.53	0.24	0.049	0.4 (0.0; 0.7)	0.645
Thin 10 ml	2.5 (0.6) 2.5 (1.5; 3.5)	2.4 (0.5) 2.5 (1.5; 3.5)	0.0 (− 0.3; 0.2) 0 (− 1; 1.5)	0.87	2.5 (0.4) 2.5 (2; 3.5)	2.5 (0.5) 2.5 (2; 4)	0.0 (− 0.2; 0.3) 0 (− 1; 1)	0.99	0.81	0.77	0.78	0.1 (− 0.3; 0.4)	0.124
Thin 20 ml	2.5 (0.5) 2.5 (2; 3.5)	2.6 (0.5) 2.5 (1.5; 3)	0.0 (− 0.2; 0.3) 0 (− 1; 1)	0.86	2.6 (0.5) 2.5 (2; 4)	2.6 (0.5) 2.5 (2; 4)	0.0 (− 0.3; 0.3) 0 (− 1; 1)	0.81	0.56	0.90	0.79	− 0.1 (− 0.4; 0.3)	0.115
Biscuit	3.6 (1.1) 4 (2; 5)	3.3 (1.1) 3.5 (1; 5)	− 0.1 (− 0.5; 0.3) 0 (− 1.5; 1)	0.59	3.5 (1.0) 3.5 (2; 5)	3.4 (1.0) 3 (2; 5)	− 0.1 (− 0.5; 0.3) 0 (− 2; 1.5)	0.81	0.95	0.85	0.91	0.1 (− 0.5; 0.6)	0.061
Residue in pyriform sinus^‡§^													
Thick 5 ml	2.0 (0.6) 2 (1; 3)	1.9 (0.5) 2 (1; 3)	− 0.1 (− 0.3; 0.1) 0 (− 1; 0.5)	0.42	2.3 (0.7) 2.5 (1; 3.5)	2.1 (0.6) 2 (1; 3.5)	− 0.1 (− 0.3; 0.2) 0 (− 1; 1)	0.60	0.21	0.28	1.00	0.0 (− 0.3; 0.3)	0.033
Thin 3 ml	2.0 (0.5) 2 (1; 3)	2.0 (0.5) 2 (1; 3)	0.0 (− 0.3; 0.3) 0 (− 1; 1.5)	1.00	2.3 (0.6) 2 (1; 4)	2.1 (0.5) 2 (1; 3)	0.0 (− 0.3; 0.2) 0 (− 1; 0.5)	0.80	0.18	0.26	1.00	0.0 (− 0.3; 0.3)	0.044
Thin 10 ml	2.3 (0.5) 2 (1.5; 3)	2.2 (0.4) 2 (1.5; 3)	− 0.1 (− 0.3; 0.1) 0 (− 1; 0.5)	0.68	2.4 (0.4) 2.5 (2; 4)	2.3 (0.6) 2 (1.5; 4.5)	− 0.1 (− 0.3; 0.1) 0 (− 1; 1)	0.39	0.38	0.81	0.90	0.0 (− 0.3; 0.3)	0.083
Thin 20 ml	2.3 (0.4) 2 (1.5; 3.5)	2.3 (0.4) 2 (1.5; 3)	0.1 (− 0.1; 0.3) 0 (− 1; 0.5)	0.62	2.4 (0.4) 2.5 (2; 3.5)	2.3 (0.5) 2 (1.5; 4)	− 0.1 (− 0.3; 0.1) 0 (− 1; 1)	0.44	0.27	0.78	0.27	− 0.2 (− 0.5; 0.1)	0.376
Biscuit	2.0 (0.5) 2 (1; 2.5)	2.1 (0.7) 2 (1; 4)	0.2 (− 0.1; 0.5) 0 (− 1; 2)	0.38	2.3 (0.9) 2 (1; 4.5)	2.3 (0.8) 2 (1; 4.5)	− 0.1 (− 0.4; 0.3) 0 (− 1.5; 1)	0.76	0.29	0.32	0.33	− 0.2 (− 0.7; 0.2)	0.338
Swallowing Performance Scale^‡§^	3.7 (1.1) 3.5 (1.5; 6)	3.4 (1.2) 3.5 (1.5; 5.5)	− 0.3 (− 0. 7; 0.1) 0 (− 2.5; 1.5)	0.19	4.0 (1.4) 3.75 (1.5; 7)	3.8 (1.3) 3.5 (2; 7)	− 0.2 (− 0.7; 0. 4) 0 (− 3; 2.5)	0.54	0.65	0.54	0.83	0.1 (− 0.6; 0.8)	0.084
EAT-10^‡^	14.2 (10.3) 14 (0; 32)	10.1 (8.1) 8 (0; 29)	− 4.1 (− 6.9; − 1.3) − 3 (− 22; 8)	0.0046	14.0 (9.6) 11.5 (1; 33)	12.5 (9.9) 10.5 (0; 31)	− 1.5 (− 3.4; 0.6) − 1 (− 8; 13)	0.17	0.98	0.52	0.13	2.6 (− 0.7; 6.0)	0.459

^*^The confidence interval for the mean difference within and between groups and *p* values are based on Fishers non-parametric permutation test

^†^Effect size was calculated as absolute difference in mean divided by the pooled standard deviation. Cohen’s standard criteria: trivial (0 to < 0.2), small (0.2 to < 0.5), moderate (0.5 to < 0.8), and large (≥ 0.8)

^‡^For secretions before swallow (1–4), initiation (1–4), residue (1–5), and EAT-10, a higher value indicates worse function. For the Swallowing Performance Scale (1–7), a higher score indicates worse swallowing function. Grades 5–7 indicate dysphagia with aspiration

^§^All ratings are reported from two raters; if there was a discrepancy between the raters, the mean of the ratings is reported; therefore, the measurements can assume values between whole scale steps

### Patient-reported outcome

No statistically significant differences between the groups in EAT-10 results were observed at any occasion (Table 5). Within-group analysis showed a significant improvement of the subjective assessment of swallowing function using the EAT-10 questionnaire at follow-up in the intervention group (mean change − 4.1, *p* = 0.004), but not among the controls (mean change − 1.5, *p* = 0.17). The difference between the changes within the groups yielded no statistical significance and a small effect size (0.46). According to suggested threshold values for the EAT-10, indicating the prevalence of dysphagia (≥ 3 points), a total of 83% of the patients (*n* = 39) experienced dysphagia at baseline (*n* = 18 in the intervention group, *n* = 21 in the control group). At the 8-week follow-up, a total of 79% (*n* = 37) experienced dysphagia (*n* = 19 in the intervention group, *n* = 18 in the control group).

### Intra- and inter-rater reliability

The intra-rater reliability for each of the two judges demonstrated substantial to almost perfect agreement of the primary outcome variable PAS (kw [weighted kappa] = 0.81–1.00). The percent exact agreement (PEA) within raters was 84–100%. The consistency of the PAS scorings between the judges was moderate to substantial for thin liquid (kw = 0.6–0.7, PEA 59–69%), substantial for thick liquid (kw = 0.7, PEA = 83%), and moderate for biscuit (kw = 0.57, PEA = 68%).

## Discussion

This randomized study aimed to evaluate the effects of an 8-week intervention program with the Shaker HLE in a HNC population using FEES and self-evaluation of swallowing. To the authors’ knowledge, no other study has evaluated the effect of the HLE using FEES measurements. The results demonstrated no improvement of oropharyngeal swallowing function assessed with FEES following 8 weeks of intervention. However, the patients in the intervention group reported subjective improvement of eating and swallowing function after HLE treatment. The assumption that treatment with the Shaker HLE is beneficial for patients with dysphagia after HNC treatment thus appears ambiguous.

Radiotherapy induces neuromuscular injury which may lead to muscular weakness and atrophy [50]. Other factors likely to affect swallowing function after oncologic treatment are tissue stiffness due to fibrosis or sensory impairment due to neuropathy [50]. As many as up to 70% of patients with HNC have been found to present with some degree of fibrotic tissue from 3 months following radiotherapy and onwards [51]. Frequently reported physiological swallowing deficits following treatment for HNC are reduced laryngeal excursion, base-of-tongue dysfunction, reduced pharyngeal contraction, impaired epiglottic movement and reduced UES opening [4, 52, 53]. As a consequence, aspiration or penetration and pharyngeal residue are commonly reported in the HNC population [4, 8, 54, 55].

The rationale for the HLE treatment is to increase swallow efficiency indirectly by increasing the strength of suprahyoidal, thyrohyoid, and pharyngeal muscles and improving the UES opening [20, 24–26, 28]. With this in mind, it was hypothesized that improvements following the HLE would be found regarding penetration/aspiration, residue, and overall swallowing function. In the present study, secretion before swallowing and initiation of swallowing were included in the evaluation as well, to possibly capture all aspects of swallowing function, not only the parts expected to change following the HLE. However, the present study demonstrated no convincing evidence for this hypothesis in the patient cohort in question, since few statistically significant differences were found in the variables assessed in FEES.

Even though the HLE may improve some of the most prevalent difficulties following HNC, such as laryngeal excursion, pharyngeal contraction, and UES opening, the exercise may have less impact on base-of-tongue function. This could at least in part explain why, even though the participants complied well to the prescribed exercise dose, there was no obvious effect on swallowing function after HLE treatment. The present results thus indicate that the Shaker HLE might not be the right type of exercise for dysphagia following oncologic treatment for HNC.

Only a few studies have previously investigated the specific effect of HLE therapy following oncologic treatment for HNC [20, 27]. They concluded that the HLE led to less aspiration [20] and shortening of the thyrohyoid muscle [27]. These results differ from the present study, where no changes regarding aspiration of any consistency was found following the HLE. The difference in result may be due to differences in patient selection, where the studies by Logemann et al. and Mepani et al. included HNC patients mixed with patients with stroke [20] or esophageal sphincter (UES) dysfunction [27]. No specific data on the HNC subgroups were presented by these authors, presumably due to the limited number of participants in the study. Therefore, it is not possible to draw any conclusions in comparison to the findings in the present study, where no changes regarding aspiration of any consistency were seen following the HLE.

Several studies have included the Shaker HLE in a battery of exercises used as preventive exercise before or during oncologic treatment for HNC [16, 21, 56–58] resulting in diverging results. As preventive exercise, the HLE together with several other exercises during oncologic treatment has resulted in improved hyoid movement, UES opening and shortening of the thyrohyoid muscle [21], better self-perceived swallowing [16], a higher rate of tolerating oral intake, and a lower extent of feeding-tube placement during oncologic treatment [56, 57]. On the other hand, no effect on swallowing safety as measured by PAS was found [58]. Since the HLE was performed together with several exercises, it is impossible to conclude which exercise was responsible for the change. However, some of the reported findings from using the Shaker HLE in combination with other exercises in a preventive manner were similar to the results of the present rehabilitation study, i.e., no improvement of PAS and some improvement of self-perceived swallowing.

The specific effect of HLE on swallowing function and physiology has also been studied in stroke patients [22, 23, 59], subjects with abnormal UES opening [24], and in healthy adults [25, 26, 60, 61]. In stroke patients, improved PAS score and swallow efficiency on modified barium swallow was noted after HLE therapy [22, 23, 59]. The UES opening during swallowing increased after HLE training both in subjects with abnormal UES opening [24] and in healthy subjects [25, 26]. Patients with abnormal UES opening have been reported with less post-swallow aspiration and a return to oral feeding instead of feeding tube following 6 weeks of HLE [24]. In the present study, only a few participants were feeding tube dependent at baseline, and in contrast to the study by Shaker et al. [24], no changes in feeding tube status occurred during the HLE intervention. It is not possible to evaluate UES function with FEES which was used in this study. Data on UES opening during swallowing in the present cohort is described in a prior study using VFSS [30]. In comparison to normal UES opening during swallowing [62, 63], the UES opening was abnormal both in the intervention and control groups at baseline in this study. Furthermore, no statistically significant differences between the intervention and control groups regarding UES opening were found after 8 weeks of treatment. Since sufficient UES opening is crucial for swallowing, this may be a part of the explanation to why the overall swallowing function did not improve as hypothesized after HLE in this patient group.

A majority (83%) of the participants in the present study reported dysphagia at baseline (i.e., ≥ 3 points on the patient-reported outcome instrument EAT-10). This is a larger proportion than recently found in a survey of a general HNC population following treatment, where 55% of their cohort experienced dysphagia following HNC [64]. One reason for this may be that our study only included patients who presented with dysphagia to some extent during the initial evaluation with VFSS. The results of the EAT-10 revealed a statistically significant improvement after HLE in the intervention group. This could be due to actual improvement of the swallowing, such as reduced residue or aspiration, but since there was no clear improvement on the instrumental evaluation of swallowing function with FEES, it is more likely that the subjective improvement may be due to other factors. One factor could be that the HLE group had more frequent contact with the SLP during the intervention. It is possible that the more frequent interaction with the SLP made the patient more confident during meals. In order to rule out this potential bias, the control group would have needed to receive equal amount of contact with the SLP, which was not the case in the present study. This could be considered a limitation and should be considered in further studies.

An asset of the present study is that it investigated the potential effect of Shaker HLE as a single, particular treatment modality together with self-perceived report of swallowing function in a randomized manner. It is, to the best of our knowledge, the only study using FEES as an outcome measure for evaluation of the effect of the Shaker HLE in HNC patients. The patients were compliant to the recommended treatment, and the HLE was monitored continuously during the intervention. The ratings of the FEES evaluation demonstrated moderate to almost perfect agreement within and between the judges. The reliability of ratings of PAS was good in this study, despite the notion that not all the videos of the FEES examinations were optimal. This indicates that PAS is useful not only for radiologic swallowing examinations (modified Barium swallow), but also for FEES.

A limitation of the study may be that the randomization process was based on the measurement of PAS using VFSS, which was not the method for evaluation of swallowing function in the present study. However, the PAS results at baseline in the present study were quite similar to previously reported PAS values in the prior VFSS study [30]. The inclusion criteria of PAS ≥ 2 led to inclusion of patients with a range of difficulties from mild to severe. The reason for this was to include patients with mixed degrees of dysphagia to reflect the variety among HNC patients in clinical swallowing practice. The inclusion of participants with PAS ≥ 2 and the exclusion of the participants with the most severe difficulties, who could not perform a swallow at all, may be a limitation, where the patients with the most severe difficulties were excluded, and participants with the least impairment were included. This possibly skews the degree of difficulties towards less impairment in total and, therefore, cannot conclude whether HLE could be helpful for those with the most severe difficulties. The randomized allocation to the different study groups was based on the PAS values together with clinical characteristics (e.g., tumor type, tumor stage, age, sex, and comorbidity) in a pursue to make the study groups as similar as possible for comparison. The possible effect of HLE treatment in defined subgroups with HNC (e.g., with different degrees of dysphagia and different types of tumors) should be addressed in future studies. The study is further limited by the number of dropouts, leaving only a total of 47 participants in the analysis. However, the comparison of the dropouts and the patients included in the analysis did not reveal any statistically significant differences, which may be considered a strength. The power analysis made before study start revealed the need of a sample size of 50 participants eligible for analysis. This study included almost 50 participants in the analysis, but it is possible that a larger sample size would have resulted in statistically significant results. Another limitation may be that no adjustments were made for multiple comparisons. However, because of the few statistically significant values yielded, the differences may be due to chance; therefore, results should be interpreted with caution. The present study did not include direct measures of muscle activity related to the HLE. For future studies, it would be of interest to include measures of muscle activity and change of muscle strength related to the HLE, in order to fully explain the effect or lack of effect, following the HLE for this patient group.

## Conclusion

Patients treated with radiotherapy for tumors of the tonsils, base of tongue, larynx, and hypopharynx did not present with improved swallowing outcome measures as assessed with FEES following 8 weeks of intervention with the HLE. Self-perceived swallowing function improved somewhat in the intervention group treated with HLE. The findings of the study indicate that the HLE alone cannot be considered an effective rehabilitation effort in patients with mild to severe dysphagia following oncologic treatment for HNC.

## Data Availability

The datasets generated during and/or analysed during the current study are not publicly available due to privacy/ethical restrictions.
